# Multimodal image registration of the scoliotic torso for surgical planning

**DOI:** 10.1186/1471-2342-13-1

**Published:** 2013-01-04

**Authors:** Rola Harmouche, Farida Cheriet, Hubert Labelle, Jean Dansereau

**Affiliations:** 1 École Polytechnique de Montréal. 2500, chemin de Polytechnique, Montreal, H3T 1J4, Canada; 2 Hôpital Ste-Justine 3175, Chemin de la Côte-Sainte-Catherine, Montreal, H3T 1C5, Canada

## Abstract

**Background:**

This paper presents a method that registers MRIs acquired in prone position, with surface topography (TP) and X-ray reconstructions acquired in standing position, in order to obtain a 3D representation of a human torso incorporating the external surface, bone structures, and soft tissues.

**Methods:**

TP and X-ray data are registered using landmarks. Bone structures are used to register each MRI slice using an articulated model, and the soft tissue is confined to the volume delimited by the trunk and bone surfaces using a constrained thin-plate spline.

**Results:**

The method is tested on 3 pre-surgical patients with scoliosis and shows a significant improvement, qualitatively and using the Dice similarity coefficient, in fitting the MRI into the standing patient model when compared to rigid and articulated model registration. The determinant of the Jacobian of the registration deformation shows higher variations in the deformation in areas closer to the surface of the torso.

**Conclusions:**

The novel, resulting 3D full torso model can provide a more complete representation of patient geometry to be incorporated in surgical simulators under development that aim at predicting the effect of scoliosis surgery on the external appearance of the patient’s torso.

## Background

Idiopathic scoliosis is a disease characterized by a complex three-dimensional curvature of the spine and the rib cage; these internal curvatures are externally manifest as a lateral trunk asymmetry and/or a rib hump. Such external deformations are often aesthetically undesirable for patients and can cause psychological problems, and in more severe cases, chronic back problems or pulmonary problems [1]. Treatments aim at slowing down the curvature progression or at correcting some of the undesired curvature. They include a brace in less severe cases and surgery in the form of vertebral fusion in more severe cases. Surgeons rely on their experience and intuition in order to establish the adequate instrumentation that would lead to the desirable post-operative external trunk appearance. However, the effects of the brace or surgical instrumentation on the external shape of the trunk cannot be reliably predicted prior to completion of treatment. Our group has recently developed a simulator with the aim of predicting the effect of scoliosis surgery on the spine and the torso [2]. Interesting preliminary results were obtained. However, the simulation outcome lacked generalizability across patients. Current research aims to integrate both bone and soft tissue in the 3D model of the trunk in order to verify whether surgical simulators that model soft tissue information could be useful in improving the prediction of the effects of surgery on the external appearance of the patient’s trunk. Such a model would require fusion of soft tissue information, typically obtained from MRIs in prone position, spine and rib cage data, typically obtained from X-rays in standing position, and a representation of the external surface of the trunk, obtained from an active vision system. This registration task is not trivial because of a mixture of both rigid and non rigid deformations that occurs between the acquisition of the different image modalities, in particular between the MRI and the remaining modalities, since the patient is lying down during MRI acquisition. Adding to the difficulty is the lack of correspondences between the tissue visible on the MRI and the structures visible on the remaining modalities. The aim of the present work is to register MRI data acquired in prone position with X-ray and TP data acquired in standing position, all while compensating for the postural changes that occur between the acquisitions and while preserving the rigidity of bone structures, in order to obtain a 3D representation (incorporating bone structures, trunk surface, and soft tissue information) of the torso of a patient with scoliosis.

Multimodal medical image registration has been applied to several types of images such as MRI/X-ray, MRI/CT, CT/X-ray, MRI/ultrasound, etc. Registration techniques can either be rigid, affine, semi-rigid, or deformable. Rigid or affine registration techniques apply either a rigid or an affine transformation to the entire source image being registered with a target image. Early work used rigid registration techniques in order to register MRI/CT and MRI/PET data of the brain [3]. In terms of non-rigid methods, several techiniques have been used in medical image registration. Those consist of thin-plate spline [4], free-form deformations using B-splines [5-8], elastic models [9], fluid models [10-13], and Markov random field approaches [14-16].

Most of the work on MRI/X-ray registration was not focused on the spine and consisted mainly of rigid registration methods [17-25] to the exception of [26] which use perspective transformations and [27], which use free-form deformations. A review of these techniques can be found in [28] and [29].

Rigid registration techniques cannot capture the complex deformation that occurs in the shape of the spine between the standing and prone positions in which the different image modalities are acquired. Furthermore, with the knowledge that the vertebrae themselves are rigid structures, traditional purely non-rigid registration algorithms are also not appropriate for the task at hand. For example, Skerl et al. [7] perform registration of vertebrae using MR and CT images, but do not model the rigidity of these structures. As a compromise, semi-rigid techniques have been used for the registration of spine data. For example, vertebral structures extracted from MRI data have previously been modeled as rigid bodies for registration purposes [30]. Soft tissue was registered using modified thin-plate splines that allow segmented vertebral structures to be constrained to rigid deformations. Similar work was done by Rohr [4], requiring only a few correspondence points instead of a full segmentation of the rigid structures. Huesman et al. [31] register CT and MR images of the torso using thin-plate spline transformations. Vertebral rigidity constraints were incorporated into the thin-plate spline approximation parameter. However, acquiring CT data for the entire torso is not possible in clinical settings due to radiation issues. More importantly, CT and MR images are both acquired in prone position, which implies that non-rigid deformations due to posture changes are not taken into account. Loeckx et al. [8] use B-splines in order to register PET/PET and CT angiography/CT angiography while incorporating a rigidity constraint in the cost function. Wang et al. [32] use triangular B-splines to deform 3 sagittal 2D MR images of vertebrae by placing knots at the boundaries of segmented vertebrae in order to insure their rigidity. B-spline functions have the advantage of allowing multiresolution registration. However, they require a regular grid of image data, which is not available in the image modalities that we wish to register. None of the works above register MRI and X-ray data, and, since all the images are acquired in prone position, nor do they model the spinal deformation between postures. Some works that have registered MRI to X-ray spine data have either required fiducials on cadaveric data [33] or required 9 X-ray images per patient for adequate X-ray/ MRI registration [34]; both strategies which are not possible in clinical settings. In later work, Van de Kraats et al. [35] generate CT-like data from MRI in order to perform rigid registration of vertebral bodies from X-ray images of cadavers. Markelj et al. [36] use 2 2D X-rays in order to rigidly register MRI and X-ray images by matching 3D gradients of 3D images to 3D gradients reconstructed from the 2D X-ray images. Tang et al. [37] apply rigid registration to fit a model of the vertebrae only onto Sagittal MRI slices in order to measure spinal curvature. Harmouche et al. [28] used an articulated model representation of the spine in order to register the spine extracted from MRI to that obtained from X-ray data of the same patient. The remaining tissues on the MRI were not registered. Finally, to our knowledge, no previous works address the simultaneous registration of MRI, X-ray and TP data.

This paper proposes a method that registers MRI to both TP and X-ray data in order to construct a 3D model covering the thoracic and lumbar vertebral levels of the torso of a scoliotic patient. MRI slices are acquired in prone position and X-ray and TP data are acquired in standing position. External markers are placed on the patient’s skin prior to X-ray and TP acquisition in order to register the two modalities using thin-plate splines. The MRI is then registered to the TP and X-ray data using a two step process: first, bone structures guide an articulated model representing the 3D spinal deformations that occur between the two postures and serves as an initial transformation of the MRI data. This transformation is then refined by confining the soft tissue to the volume delineated by the trunk surface and the surface of the vertebrae using weighted thin-plate splines with constraints for bone rigidity and soft tissue elasticity. The availability of the surface contours allows us to model the rigidity constraint in a simpler fashion when compared to previous works. To our knowledge, this is the first work that combines bone, soft tissue, and surface topography information for a model of the human torso.

This article is separated as follows: The Methods section describes the data acquisition process and the articulated model based registration. Qualitative and quantitative results of the proposed method are compared with both rigid and simple articulated model registration in the Results and discussion section. The key conclusions and proposed future works are presented in the Conclusions section.

## Methods

In order to obtain the 3D representation of a patient incorporating bone, soft tissue and surface information, MRI, X-ray images and TP data are obtained for scoliosis patients. The 3D spine models are then extracted from the MRI and X-ray images and the 3D positions of external markers are obtained from the TP and X-ray data. The goal is to then align MRIs of the torso with reconstructed thoracic and lumbar vertebrae from biplanar X-ray images and surface topography data (TP). Thus, we are searching for the independent transformations *T*_*TP*−*Xray*_ and *T*_*MRI*−*Xray*_, which would transform MRI and TP data into X-ray space, respectively.

### Data acquisition and preprocessing

MRI, X-ray images and TP data are obtained for three adolescent patients with scoliosis at the Sainte-Justine Hospital in Montreal. Patient 1 is 153.4 cm tall, weighs 48.8 kg, and has a right thoracic curvature with a 50° Cobb angle. Patient 2 is 146.1 cm tall, weighs 41.7 kg, and has a left thoraco-lumbar curvature with a 57° Cobb angle. Patient 3 is 160.5 cm tall, weighs 46.2 kg, and has a right thoracic curvature with a 49° Cobb angle. The present research involving human subjects has been approved by the ethics committee of the CHU Sainte-Justine Research Center (Comité d’Éthique de la Recherche du CHU Sainte-Justine). Written consent is obtained from the participants and a parent or guardian. These 3 image modalities are obtained during the same day but at different times for each patient.

**Figure 1:**
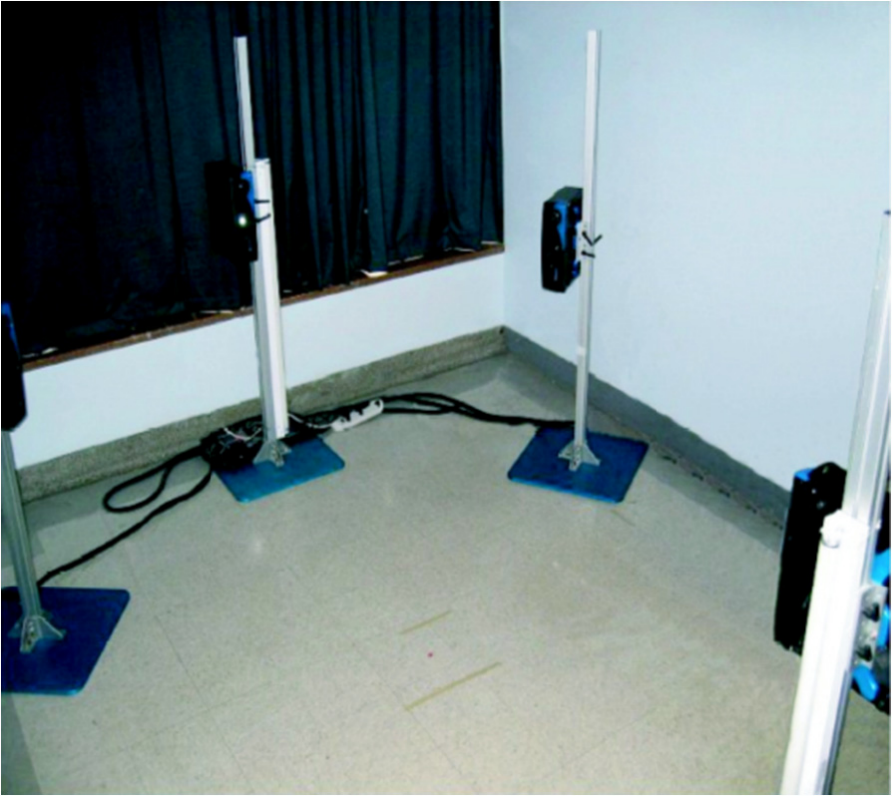
**Inspeck system.** Inspeck system used for capturing surface topography data. Four digitizers allow the capturing of the full surface of the patient torso.

**Figure 2:**
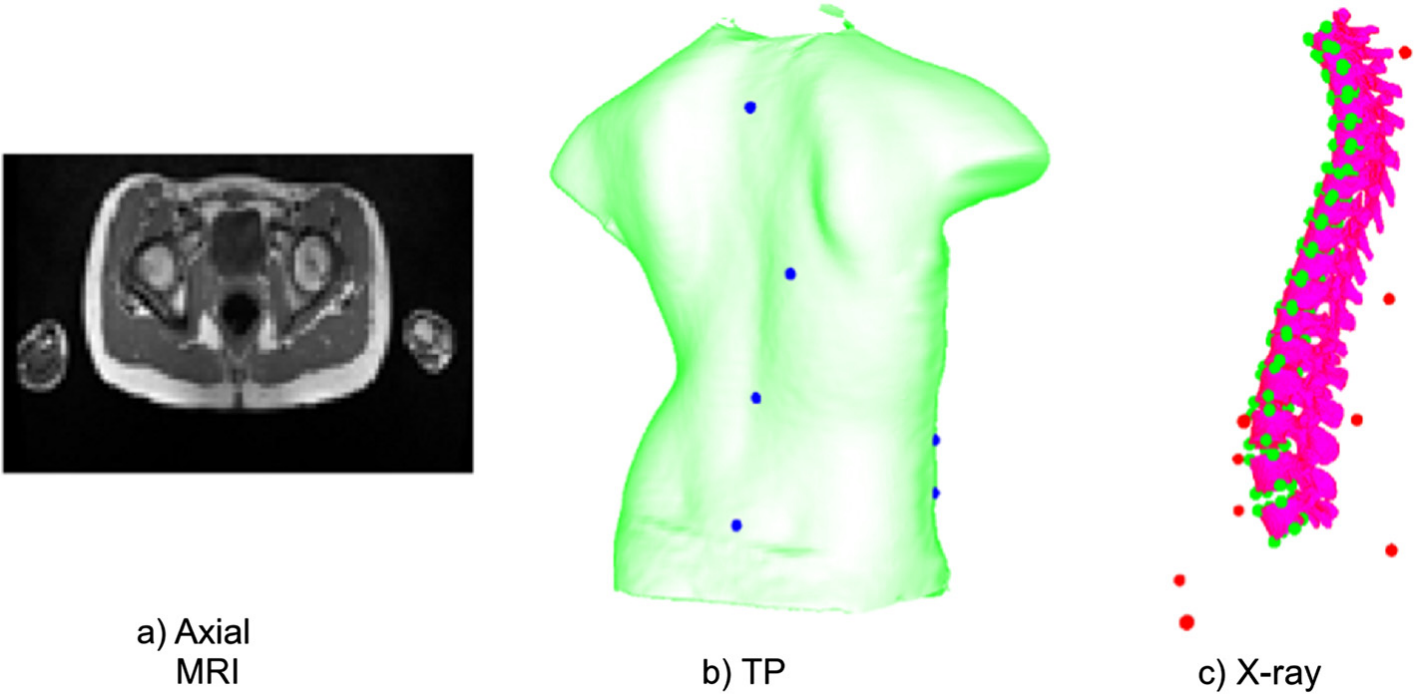
**Medical image modalities.** Multimodal image data used for the 3D patient representation developed. The spine representation in (c) is reconstructed from bi-planaer X-rays. The externally placed landmarks are displayed in blue on the surface topography and in red on the X-ray image. The vertebral landmarks used to construct the articulated model are displayed in green on the X-ray (c).

First, four 3D digitizers (Creaform inc., average resolution of 1.1 mm), each covering a different view of the patient, are used in order to obtain the surface topography of each patient’s torso (Figure 1). Each digitizer consists of a color CCD camera and a structured light projector. The patient is instructed to stand in the middle of the camera setup, legs slightly apart, arms slightly raised to the sides. This posture was found to minimize occlusions to the patient’s torso all while maximizing patient comfort (thus minimizing patient movement). Deformed patterns are then obtained from each digitizer and are used to retrieve shape and texture information. The shape information obtained from the different views is merged using the Inspeck EM software resulting in a mesh representation of the surface topography (Figure 2(b)). Prior to acquisition, several visible green adhesive markers are placed on the surface of the patient’s torso. The markers are placed at the following locations: vertebra prominens, pilonidal dimples, antero-posterior iliac spine, sternum, spinous process, and inferior angle of the shoulder blades. These markers are regularly used in our clinical setting since their localisation was found to be the most reproducible. The 3D position of the adhesive markers is then obtained by manually identifying the markers on the digitized texture information using in-house software.

Postero-anterior and lateral radiographs are then obtained for each of the patients while they are in standing position, legs slightly appart, and arms to the front and bent upwards. An explicit calibration method [38] is then used in order to calculate the 3D position of vertebral landmarks manually identified by experts on both X-rays. The obtained landmarks allow the mapping of generic vertebral surface meshes onto the patient space; resulting in a patient-specific 3D geometric model of the spine from the X-ray data (Figure 2(a)). In this case, six of those landmarks are used per vertebra in order to generate the X-ray articulated model. These are placed on the centers of the superior and inferior plates of each of the 17 thoracic and lumbar the vertebral bodies, and below and above the left and right pedicles. The chosen landmarks are regularly used for vertebral reconstruction in our clinical setting since they are clearly visible on the X-rays. In addition, prior to image acquisition, radio-opaque markers which are clearly identifiable on the obtained X-rays are placed on the surface of the patient’s torso and affixed on top of the adhesive markers used for TP data acquisition. The 3D position of the markers is then calculated using the same methodology as for the vertebral landmarks. These external markers are used for TP/X-ray registration.

Sagittal MR images of the spine are routinely acquired in our clinical setting for scoliosis patients awaiting surgery (1.5 Tesla, TR/TE = 771/15, 704x704, 350 FOV, with a 0.5 mm by 0.5 mm in-plane resolution and 3 mm thickness, with a 3.6 mm separation between slices). In order to visualize the entire torso, T1-weighted axial MRI slices of 1 mm by 1 mm in-plane resolution, 2 mm thickness and 12 mm spacing between slices and covering the entire torso are acquired (Siemens Symphony system 1.5 Tesla, TR/TE = 650/12, 704x704) (Figure 2(c)). Since acquisition of the sagittal slices already requires a total of 30 minutes inside the MRI, the larger spacing between the axial slices allows for the entire trunk to be imaged in a reasonable amount of time for patient comfort. The time inside the MRI thus totals around 50 minutes including rest time between acquisitions. Since the sagittal and axial slices are acquired during the same scan, their location with respect to each other is known and they are thus pre-registered. The sagittal slices are thus used in order to situate the axial MRI acquisitions to the appropriate vertebral level and consequently to calculate the transformations required. This is required because the distance between the axial slices is large and the contours of the vertebrae are not all visible on these acquisitions. It must be noted that in cases where sagittal slices are not required for clinical purposes, we would be able to use the 50 minute total scan time in order to acquire the axial slices only, thus allowing for a significantly better resolution and better visibility of the vertebrae. In order to generate a 3D geometric model of the spine from MRI data, the 3D shape of the seventeen thoracic and lumbar vertebrae is manually segmented from the sagittal slices using TomoVision’s SliceOmatic software. Landmarks are then manually placed on the left and right edges of the posterior, anterior, inferior and superior ends of the vertebral body for all thoracic and lumbar vertebrae also using TomoVision’s SliceOmatic software. These landmarks are used in order to calculate the articulated model for MRI/X-ray registration.

### X-ray / TP registration

The TP data is registered to the X-ray reconstruction by applying a thin-plate spline transform (*T*_*TP*−*Xray*_) [39]. The transform has the following form:

(1) $$f(x,y,z)=a_{1}+a_{2}x+a_{3}y+a_{4}z+\overset{n}{\underset{i=1}{\sum }}w_{i}U(r),$$

where *f*(*x*,*y*,*z*) is a vector valued function which maps each point (*x*,*y*,*z*) of the original TP data onto a point (*x*^*′*^,*y*^*′*^,*z*^*′*^) in X-ray space. The *a*_*i*_ are the coefficients of the affine transformation, *w*_*i*_are the weights, and *r* is the distance between the control point and the point to be transformed. *U*(*r*)=|*r*|for a 3D transformation, as was stated in the works of Bookstein [39]. The *a*_*i*_ and *w*_*i*_ are estimated by solving the following system of linear equations using the manually placed markers on the surface of the patient prior to acquisition of both TP and X-ray data:

(2) $$\begin{array}{rll} \mathrm{Kw}+\mathrm{Pa} & =V,\;\\ P^{T}w & =0,\; & \; \end{array}$$

where *P* is the vector of source points obtained from the TP data, *V * is the vector of target points obtained from the X-ray data, and *K* is the matrix containing *U*(*r*).

### Transforming MRI data

Since the spacing between consecutive axial MRI slices is large, they are treated as independent 2D slices and the continuity between successive slices is not adressed. Thus, each slice is registered separately to the remaining model using *T*_*MRI*−*Xray*_, which a composition of an articulated-model transformation (*T*_*MRI*−*bone*_) and a thin-plate spline transformation (*T*_*MRI*−*soft*_). The goal is to transform each MRI voxel into the space of the 3D X-ray model, taking into account the non-rigid deformation following the posture change subject to the following constraints: First, the spine extracted from the MR images has to be aligned with the X-ray spine model. Second, MRI data of the torso has to be contained within the TP volume, such that the contour of the torso on the MRI corresponds to the surface topography.

In order to register each MRI slice such that the spine information extracted from MRI data and that extracted from X-ray data are aligned, an articulated model previously used to align 3D models of the spine obtained from both modalities is calculated [28]. This articulated model allows us to define the spine as a combination of local intervertebral transformations which can be obtained in a number of ways. In our case, we segment the vertebrae from the sagittal MRI data in order to extract the vertebral landmarks. The sagittal slices are used since they are readily available in our clinical setting and have a better resolution than the axial slices. The local coordinate system of each of the thoracic and lumbar MRI vertebrae *v* is then obtained by calculating the center and the orientation of the vertebrae. Similarly, the local coordinate system of each of the thoracic and lumbar X-ray vertebrae is obtained using the X-ray vertebral landmarks. Intervertebral transformations consisting of rotations and translations are then defined between the local coordinate system of each vertebra *v* and its lower neighboring vertebra *v*−1. Although landmarks are used in order to obtain the local coordinate system of each vertebra, the landmarks extracted are different for the X-ray and MRI data and thus point correspondences cannot be established. Thus, they have not been explicitly used for registration. In fact, future work consists of obtaining the local coordinate system from automatic vertebral segmentations, which can replace the need for manually placed landmarks. The global transformation for each of the vertebrae is calculated separately using a composition of the local rigid intervertebral transformations.

(3) $$T_{0,v}=T_{v-1,v}\circ T_{v-2,v-1}\circ \ldots \circ T_{1,2}\circ T_{0,1},$$

where *T*_0,1_ is the global rigid transformation between the world coordinates and the first vertebra on each of the two image modalities. The overall articulated model transformation (*T*_*MRI*−*bone*_)from MRI to X-ray space is then obtained using a concatenation of the global MRI and X-ray vertebral transformations:

(4) $$T_{v-\mathrm{MRI}-\mathrm{bone}}=T_{0,v-\mathrm{Xray}}\circ T_{0,v-\mathrm{MRI}}^{-1}.$$

**Figure 3:**
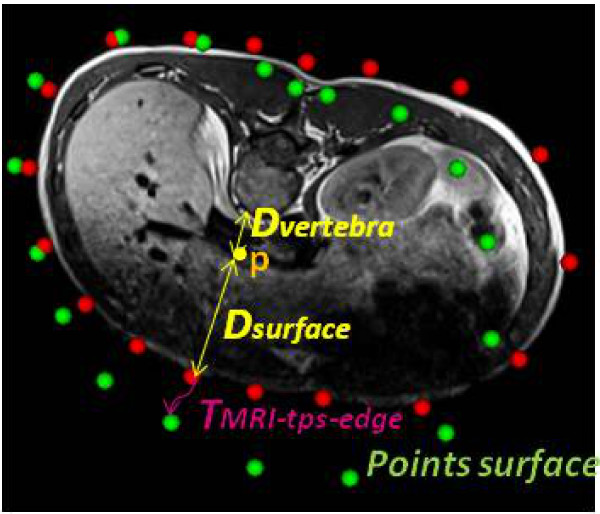
**Ratio for weighted thin-plate spline.** Calculation of the weighted thin-plate spline ratio based on the distance of the point *p* to be registed, the closest vertebra, and the surface of the torso. The points in green are obtained from the contours of the surface topography and the points in red are obtained from the MRI.

Once the transformation model between the MRI data acquired in prone position and X-ray spine acquired in standing position is obtained, it can be used to provide a preliminary registration for the axial MRI slices. Since both the sagittal and axial MRI data are acquired at once, they are already registered to the same space and thus can be used interchangeably. Each axial MRI slice *s* is transformed using the vertebral transformation *T*_*v*__*s*__−*MRI*−__*bone*_ where *v*_*s*_ is the vertebra that has the closest z value to the slice. This part of the transformation takes into account the changes in the general alignment of the MRI slices due to changes in posture. This is followed by a weighted thin-plate spline transformation *T*_*MRI*−*soft*_ in order to approximate the non-rigid deformations on each slice separately. This transformation is illustrated in Figure 3 and is calculated in the following manner: first, the plane corresponding to each of the registered MRI slices (i.e. having the same location and angle) is obtained from the surface topography data. In order to guide the non-rigid registration between the MRI and TP planes, correspondence points are extracted from the torso contour of the MRI slice (visible in red in Figure 3 ) and from the corresponding plane on the surface topography (visible in green in Figure 3). These points are extracted at 30 degree intervals, angle 0 being a vector passing from the center through the anterior point of the vertebra. The MRI slices have already been transformed to the appropriate location and angle with respect to the vertebrae following the articulated model registration. Thus, points in the same direction on a slice can be used as correspondences. These correspondences are used given the fact that we do not have access to anatomically significant landmarks and that the purpose of the correspondence points is to drive the thin-plate spline registration by ensuring that the interior of the surface topography is fully covered by the MRI slice. A preliminary thin-plate spline transformation *T*_*MRI*−*soft*−*edge*_ is approximated by solving the same system of equations as in equation 2. The points extracted from the edges of the segmented MRI are used as source landmarks and those obtained from the corresponding plane on the surface topography are used as target landmarks. The transformation of an arbitrary point *p* is then a modified version of the thin-plate spline transformation *T*_*MRI*−*soft*−*edge*_: A weight is incorporated depending on the relative distance of the point *p* from the edge of the torso and from the closest vertebra, given that this transformation should be equal to the identity matrix *I* within the vertebra. The overall transformation becomes:

(5) $$T_{\mathrm{MRI}-\mathrm{Xray}}=T_{\mathrm{MRI}-\mathrm{soft}}\circ T_{v-\mathrm{MRI}-\mathrm{bone}}$$

where, for each point *p*:

(6) $$\begin{array}{lll} \;T_{\mathrm{MRI}-\mathrm{soft}} & =I,\;p\;\mathrm{inside}\;\mathrm{vertebra},\; & \;\\ & =\frac{D_{\mathrm{vertebra}}}{D_{\mathrm{surface}}\;+\;D_{\mathrm{vertebra}}}*T_{\mathrm{MRI}-\mathrm{soft}-\mathrm{edge}},\;\;\mathrm{otherwise},\; \end{array}$$

where *D*_*vertebra*_is the distance between point *p* and the border of its closest vertebra obtained from the MRI data and *D*_*surface*_ is the distance between point *p* and the border of the segmented MRI torso. Since the meshes representing the X-ray vertebrae were originally obtained from cadaveric data and registered to patient space using a few interest points, the use of the MRI vertebral meshes were favoured in order to calculate this distance. The MRI vertebrae were segmented from real patient data. The weighting applied to the thin-plate spline at each voxel is such that the further we are from the vertebra, the more weight is given to the non-rigid deformation. The proposed transformation was chosen as a simple and sufficient way to model the deformation between the vertebrae and the torso surface, as there is a lack of correspondences between the different image modalities in that area. In addition, the present goal is to simply fill the volume contained within the surface topography with approximate soft tissue information that would allow for the creation of a 3D patient model for surgical simulations.

## Results and discussion

Following the desciption of the validation methods, qualitative and quantitative results for our registration method will be presented.

### Validation

The proposed registration method is compared to rigid registration and simple articulated registration. Qualitative results are followed by overlap comparisons between the surface contained within the torso on the TP and the registered MRI slices using the Dice similarity coefficient [40]. In order to calculate the Dice measure, a mask of the torso data is obtained for each axial MRI slice. A corresponding planar cut through the surface topography at the same position in 3D patient space and in the same orientation as the registered MRI slice is obtained and a second mask of the interior of the surface topography is then calculated. The overlap between the two masks is then calculated using the following equation:

(7) $$\mathrm{Dice}=\frac{2*(\mathrm{MRI}\cap \mathrm{TP})}{\mathrm{MRI}\cup \mathrm{TP}}$$

Higher Dice values signify better overlap between the 2 surfaces, with perfect overlap corresponding to a Dice value of 1. The assessment of Dice values is somewhat dependent on the size of the areas being compared. That is, larger areas have higher area to perimeter ratio resulting in inherently higher Dice values. Since in the present case the different methods are comparing the same area being deformed, the Dice value is adequate for the comparison. In addition, the Dice values calculated assume that the MRI slices are in the proper orientation following registration and correspond anatomically to the surface topography slice at the same location and orientation. We make this assumption as the MRI slices have been aligned using the articulated model. We tolerate any bias obtained following this assumption given the fact that our main aim is to verify whether the soft tissues fit within the space encompassed within the surface topography. The Dice measure is able to verify this aim. The mean and variance of the Dice values is calculated for all patients, in addition to testing for statistically significant differences between the Dice values of the different methods.

In order to provide a more precise Dice measure that does not depend on the orientation of the slices, a 3D Dice analysis is preformed. This analysis reduces the bias present in the 2D Dice calculations. In order to obtain a volume from the registered MRI slices, we first build a surface mesh over the entire torso by triangulating points sampled from the MRI contours using a Delaunay triangulation. Since the MRI slices have a large distance between them, a more detailed mesh is then obtained using linear subdivision. We then create a volume with a resolution of 1 mm x 1 mm x 3 mm and having slices parallel to the x-y plane, and set all voxels inside the surface to be equal to 1, otherwise 0. This resolution is chosen since it is only slightly larger than the MRI slice thickness, and such a voxel size has been successfully used for MRI segmentation applications in the past. A similar volume with the same resolution is obtained from the surface topography mesh. We then compare every voxel of the obtained MRI and TP volumes using the Dice measure.

In order to assess the quality of the deformation field resulting from our proposed registration, and to examine the areas that display the highest amount of deformation, the determinant of the Jacobian of the deformation field is calculated both with and without the weighting applied on the thin-plate spline transformation. The Jacobian of the deformation field *J* is defined as the matrix of partial derivatives of the deformation field. It has been used in previous works on image registration in order to ensure a smooth and topology preserving transformation [41]. For each voxel, the transformation vectors are calculated using the methods described previously. The discrete partial derivatives making up the Jacobian are then approximated using the first order derivative of a Gaussian (sigma = 1). The determinant of the Jacobian is obtained from the partial derivatives and indicates the voxel-wise relative volume change. For example, a determinant value of 3 indicates that the original volume has decreased by a factor of 3. In order to insure volume preservation of the tissues and organs during registration, a determinant value of 1 is required. The determinant of the Jacobian for the deformation of all voxels of MRI slices are shown and areas of highest deformation are addressed. The mean and variance values for all slices are then calculated.

### Qualitative results

**Figure 4:**
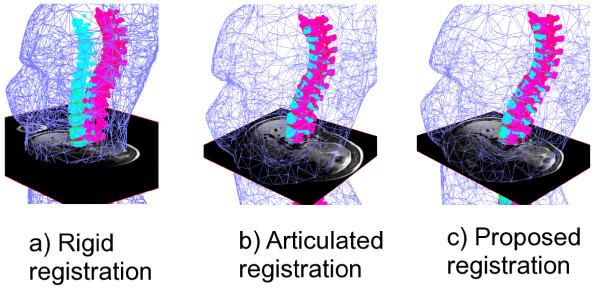
**Registration results.** Results comparing rigid registration with registration using the proposed method for the MRI, X-ray and TP. The X-ray vertebrae are displayed in pink and the MRI vertebrae are displayed in cyan. A decimated mesh representing the surface topography is displayed in blue. It can be seen the results with the proposed method yield a better alignment of the MRI and X-ray vertebrae and a better fitting of the MRI within the surface topography.

Figure 4 shows results for 1 slice of patient 1. A clear misalignment can be seen between the vertebrae extracted from the X-ray data and those extracted from MRI data when rigid registration is used (Figure 4(a)), illustrating the difference in the shape of the spine between the two postures in which these two modalities are acquired. As a result, the axial MRI slice displayed is misaligned to the surface topography and the X-ray vertebrae. The articulated model provides a better vertebral alignment (Figure 4(b)), but does not provide an adequate fitting between the MRI soft tissue data and the surface of the trunk. The torso on the MRI slice shows better alignment with the surface topography compared to both rigid and articulated-model registration (Figure 4(c)). The proposed method is thus able to correct for some of the deformations that can be visible on the MRI due to the change in posture between the image modalities.

### Quantitative results

#### Dice anaylsis

**Figure 5:**
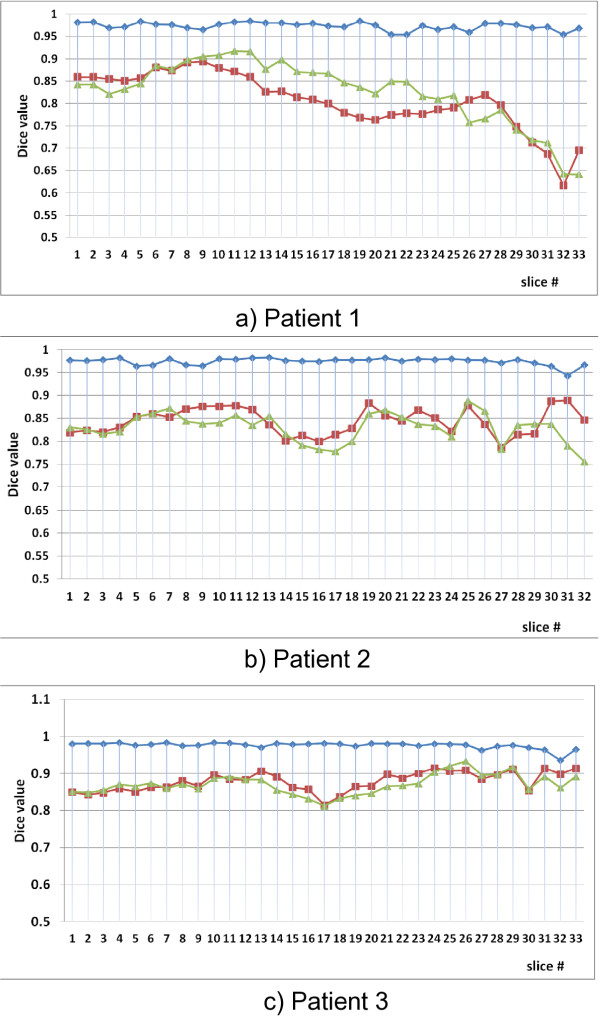
**Quantitative registration results.** Dice values for patients 1,2 and 3 measuring the overlap between torso data obtained from axial MRI slices and corresponding surface topography axial cuts for all patient slices for rigid (red), articulated (green) and the proposed (blue) registration methods. Results range from inferior to superior axial slices.

Figure 5(a) presents a graph of the Dice values for all 33 axial slices of patient 1 covering the thoracic and lumbar vertebral levels. An average Dice value of 0*.*973±0*.*008 is observed for the proposed method compared to 0*.*806±0*.*064and 0*.*826±0*.*071 for the rigid and articulated registrations, respectively. A statistically significant improvement is observed when the proposed method is used compared to both the rigid and the articulated model registration (*p* < 0*.*01 in both cases). A statistically significant improvement is also observed when the articulated model registration is compared to rigid registration (*p*<0*.*01). The articulated registration performs worst on slices containing breast tissue (slices 15 onwards), where our method is better able to approximate the non-rigid deformations of the breasts. Our proposed results still contain inaccuracies in the breast area as their deformation is somewhat independent from the rest of the tissues. However, proper modeling of breast deformation was not addressed in the present work as it does not affect the accuracy of the outcome of a scoliosis surgery.

Figures 5(b) and 5(c) show the Dice values for patients 2 and 3, respectively. A significant improvement in Dice values is also observed in these cases as well when our method is compared with rigid and articulated registrations. For the case of patient 2, the average Dice values are 0*.*844±0*.*028, 0*.*830±0*.*031 and 0*.*974±0*.*008 for rigid, articulated, and proposed registrations, respectively. A statistically significant improvement is also observed in the case of patient 2 when the proposed method is used compared to both the rigid and the articulated model registration (*p*<0*.*01 in both cases). However, an improvement was not seen when rigid and articulated model Dice values were compared.

In the case of patient 3, the average Dice values obtained are 0*.*878 ± 0*.*052, 0*.*871 ± 0*.*010, and 0*.*976 ± 0*.*010 for rigid, articulated, and proposed registrations, respectively. As in the case of patient 2, a statistically significant improvement is observed when the proposed method is used compared to both the rigid and the articulated model registrations (*p* < 0*.*01 in both cases), and no improvement is observed when rigid and articulated model registrations are compared.

The 3D Dice measure is obtained for the 3 patients using the results from the proposed method. In this case, values of 0.939, 0.938, and 0.940 were obtained for patients 1, 2, and 3, respectively. These values are only slightly lower (by an average of 3.66%) than those obtained using the 2D Dice measure. This is to be expected since, unlike the 2D Dice measure, the 3D measure compares voxels within the patient’s surface that were not used in the registration process. More importantly, these errors are inherent to the fact that an interpolation, more so a linear interpolation, was used between consecutive MRI slices due to the low MRI resolution and not to the method itself. Nonetheless, the values obtained using the 3D measure are still considered excellent in the literature, and are still considerably higher (by an average of 11.52%) than the 2D values obtained for rigid and articulated registration. We can thus conclude that, although the spacing between consecutive MRI slices is high, our method still provides a volume of soft tissue information contained within the surface topography.

One obvious caveat with the Dice similarity coefficient to validate our work is that it does not measure the anatomical correctness with which our soft tissues were deformed. However, for the desired precision, given that the MRI resolution is low in the z-direction thus leading to a less precise overall model, that a measurable difference in trunk shape changes due to treatment is in the order of a tenth of a cm, and that no anatomical correspondences are present in the space contained between the vertebrae and the patient’s trunk surface, the current results are deemed satisfactory. Our main concern is to obtain a model with which the vertebrae maintain their rigid characteristics and the space contained within the surface of the patient is filled with soft tissue information. However, we must note that the presented registration framework does not take the physical characteristics of the tissues into account when modeling the deformations.

#### Assessment of the transformation

**Figure 6:**
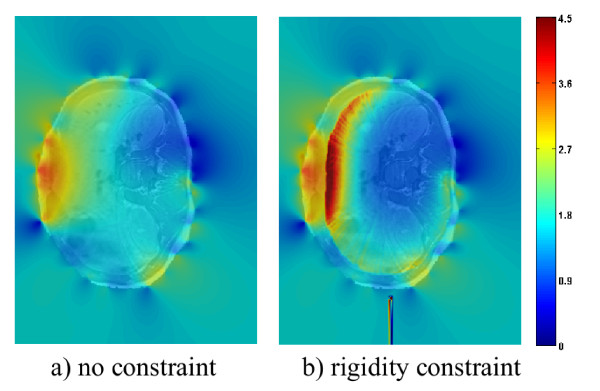
**Determinant of the Jacobian for slice 9.** Determinant of the Jacobian of the deformation field resulting from registration without **(a)** and with **(b)** rigidity constraints for slice 9 of patient 3. A spectral intensity mapping is displaying |*Jacobian*(*i*)−1|for pixel *i* superimposed on top of the registered MRI slice. The spectral mapping shows values closer to 1 as blue and values further from 1 as red. The anterior portion of the torso is to the left of the image.

The quality of the transformation is studied using the determinant of the Jacobian. Figure 6 shows the determinant of the Jacobian of the deformation field overlayed on top of the MRI data for slice 9 of patient 3, which is situated around the stomach area. Results without (a) and with (b) the rigidity constraint are displayed. The spectral mapping shows values closer to 1 as blue and values further from 1 as red. Since we did not impose any requirements on volume preservation, the fact that that most values are far from 1 in (b) is not problematic. However, it is interesting to see which areas display the most significant amounts of variation in the deformation. Both cases have higher Jacobian values towards the anterior portion of the torso (left of the images). This is because there has been an increased amount of volume expansion around the stomach following registration, which was intended to counter tissue compression that occurrs while the patient is lying down during MRI acquisition. The main difference between the values with and without the rigidity constraint is that, in the case of our proposed method, the determinant is equal to one inside the vertebra (due to the rigidity constraint imposed), and increases as voxels go radially outwards towards the surface of the patient’s skin, since a higher weight is placed on the non-linear portion of the deformation.

**Figure 7:**
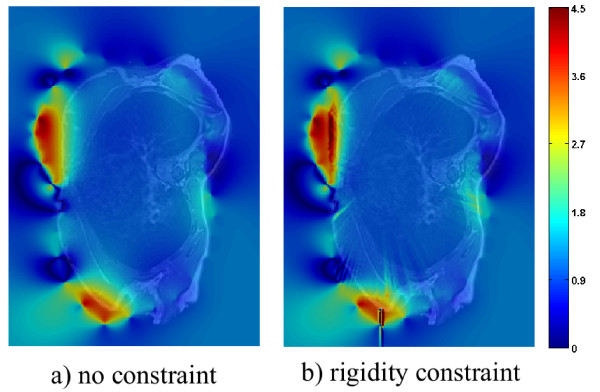
**Determinant of the Jacobian for slice 25.** Determinant of the Jacobian of the deformation field resulting from registration without **(a)** and with **(b)** rigidity constraints for slice 25 of patient 3. The intensity mapping is the same as in the previous figure.

Figure 7 shows the determinant of the Jacobian of the deformation field for slice 25 of patient 3, which is situated around the breast and shoulder area. In each of these images, the red and yellow areas are located in the breast region. In this case, deformation varies much less smoothly around the breasts than in the remaining areas. These results are expected, as the breasts are likely to deform independently from the articulated model used.

### Discussion

The results above show that the proposed method is able to register the MRI, X-ray and TP data of a human torso with satisfactory precision, and doing so while still compensating for the deformations that occur between images due to differences in posture in which these images are acquired. The residual registration errors still present in the results may be due to several factors. For example, the precision of the manual intervention required for the localization landmarks on all images has inherent limits. X-ray and MRI landmark localisation errors have been studied in previous works to be 2*.*1±1*.*5 *mm*[42] and 3*.*17±3*.*3 *mm*[28]. The MRI landmark localisation errors have been shown to significantly decrease to 1*.*57±1*.*13 *mm* when the centroids are compared, as is the case for our proposed registration method. In the case of the surface topography, where the resolution of the equipment is 1*.*1 *mm*, the landmark localisation error is assumed to be equivalent to the radius of the adhesive markers, thus 2*.*5 *mm*. The registration error between the surface topography and the X-ray data has been previously established at 2*.*7 *mm*[43]. Ongoing work within our group is aimed at automating the landmark extraction process. Furthermore, unlike existing methods, our proposed articulated model method does not require actual correspondence points for registration. This is due to the fact that the center and the orientation of the vertebral bodies are used in order to calculate the transformation between vertebrae. Thus, the need for landmark extraction can be eliminated and replaced with the use of higher order primitives. This has the potential to reduce registration variability and to improve precision. In terms of the correspondence points used to drive the thin-plate spline registration between the MRI and surface topography, the accuracy is not studied. These correspondences might suffer from a lack of accuracy due to the fact that deformations in the z axis between the 2 modalities that are due to gravity have not been taken into account by the articulated model. However, a lack of anatomically significant correspondences makes it difficult to measure the accuracy. Since the landmark selection is automatic, we assume that the precision is mostly dependent on the resolution of the images. In this case, the image with the lower resolution is the MRI. The MRI resolution is 1 mm by 1 mm in-plane and has 2 mm thickness leading to a maximum distance of 2.45 mm between voxels.

Further refinement of the method can be made by incorporating tissue-specific elasticity constraints, which would require tissue-specific biomechanical analysis resulting in a more complex model. This would provide for more realistic anatomical deformations. It should also be noted that tissues contained within the boundaries of the ribcage are believed to deform differently from tissues outside of this boundary. Modeling the transformation of these tissues differently has the potential of improving registration precision. In addition, a thorough biomechanical analysis would allow us to model the effect of gravity on the non-rigid deformations resulting from a posture change. The implication is that gravity would have a different effect on the various anatomical structures being registered. However, the 3D voxel deformations resulting from gravitational forces are very likely to be outside of the plane of the acquired MRI slices. Thus, incorporating these deformations would require a higher resolution in the z plane, which is currently infeasible in a clinical setting due to prohibitive acquisition times.

## Conclusions

A method to register MRI, X-ray and surface topography data was proposed. This method first registered surface topography and X-ray data using thin-plate splines, and then fit the MRI data onto the model by taking into account the non-rigid deformations that are due to the postural difference between acquisitions. An articulated model was used in order to approximate the vertebral deformations in the MRI, and the remainder of the soft tissues was deformed using thin-plate splines with vertebral rigidity and the surface topography as constraints. Visual results as well as 2D and 3D Dice values measuring the fit between the surface topography and MRI data were obtained for real data of 3 pre-operative patients with scoliosis in order to validate the proposed method. Both qualitative and quantitative results showed a significant improvement in fitting the MRI data with the X-ray and surface topography data when compared to both rigid and simple articulated model registration. The determinant of the Jacobian of the deformation field was obtained with and without the rigidity constraint, showing higher variations in the deformation in the anterior part of the slices, and showing lower variations closer to the vertebrae in the case of the proposed method. Future work aims at incorporating tissue-specific elasticity constraints to the registration process, and at using automatically extracted higher order primitives for the articulated model registration. The precision of the obtained registration results allows us to build a complete 3D model of a patient’s trunk including soft tissue, vertebral, and trunk surface information which can be incorporated in a surgical simulator under development in order to potentially better predict the outcome of scoliosis treatments.

## Competing interests

The authors of this work hereby declare that they have not received reimbursements, fees, funding, or salary from an organization that may in any way gain or lose financially from the publication of this manuscript, either now or in the future. They do not have any other financial competing interests to declare.

## Authors’ contributions

RH participated in the development of the methodology, gathering patient data, and carrying out experiments, results, and writing of the manuscript. FC participated in the development of the methodology and in the writing of the manuscript. HL provided patient data and participated in writing of the manuscript. JD participated in the development of the methodology. All authors read and approved the final manuscript.

## Pre-publication history

The pre-publication history for this paper can be accessed here:

http://www.biomedcentral.com/1471-2342/13/1/prepub
